# Microwave-Assisted Extraction of Pectin from Pickled Cabbage Waste: Process Optimisation, Characterisation and Application in Low-Sugar Jam

**DOI:** 10.3390/polym18182200

**Published:** 2026-09-09

**Authors:** Fatma Cebeci Aydın, Tayyibe Erten

**Affiliations:** Department of Nutrition and Dietetics, Faculty of Health Sciences, Bayburt University, 69000 Bayburt, Türkiye

**Keywords:** microwave-assisted extraction, low-methoxyl pectin, valorisation, pickled cabbage waste, characterisation, low-sugar jam, RSM, optimisation

## Abstract

This study optimised pectin extraction from pickled cabbage waste using microwave-assisted extraction (MAE), characterised the extracted pectin, and explored its potential for low-sugar jam production. The Box–Behnken design concluded that the optimal conditions are 539 W (microwave power), 116 s (time), and pH 1.5, yielding a pectin yield of 22.53 ± 2.63%. The physicochemical analysis revealed that the microwave-extracted pectin (MEP) was low methoxyl and had a high molecular weight (1.20 ± 0.004 MDa). It was composed of galacturonic acid, arabinose, rhamnose, xylose and glucose. FTIR, ^1^H NMR and SEM results also confirmed structural characteristics of MEP. MEP was confirmed to have remarkable technological properties, including an emulsion activity of 53.43 ± 2.19% and good stability over 30 days at 4 °C and 24 °C. Moreover, it has an oil-holding capacity of 1.18 ± 0.51 g oil/g pectin and an elevated water-holding capacity (9.36 ± 3.25 g water/g pectin). The application of MEP as a stabiliser in low-sugar jam enhanced viscoelastic characteristics of jam, like apple pectin, and G′ > G″ confirmed the improved shelf-life stability at 25 °C. This study emphasised that microwave-assisted extraction can be a sustainable, time- and energy-saving method for the valorisation of pickled cabbage waste, and highlighted the effect of MEP on the characteristics of low-sugar jam.

## 1. Introduction

Pectin is a well-known food additive used in food manufacturing as a gelling, thickening, or stabilising agent, or as a dietary fibre source. Although it is mainly extracted from citrus peels, growing market demand is driving innovation in pectin production and highlighting the importance of recovering pectin from agro-industrial organic waste using eco-friendly methods [1]. However, the extraction method is the key determinant of this valorisation since the conventional methods bring inconveniences such as prolonged extraction time and low yield and quality, in addition to the high volume of wastewater generated, which raises environmental pollution concerns. Therefore, novel extraction techniques, including the use of food-grade organic acids [2], deep eutectic solvents, or surfactants such as alternative solvents [3], ultrasound-assisted extraction [4,5], subcritical fluid extraction [6], ohmic heating extraction [7], and microwave-assisted extraction [8], were frequently investigated. To achieve eco-friendly production and enhanced pectin yield and quality, microwave-assisted extraction stands as an alternative method with shorter extraction time, higher yield, and a more homogeneous temperature distribution compared to conventional acid extraction [9].

Pickled cabbage is consumed all over the world as a part of the daily diet [10]. Although it is a popular fermented product worldwide, large amounts of organic waste are generated during its production. For instance, the local food factory that supplied the waste for this study experiences an average loss of 10% during the manufacturing process and quality control. This portion is considered organic waste and is not utilised in any way. With the prominence of sustainable waste management efforts in recent years, the utilization and recovery of cabbage waste have also gained importance [11,12]. Therefore, this study investigated the extraction of pectin from pickled cabbage waste using microwave-assisted extraction as an environmentally friendly method. Then, the obtained pectin was characterised in terms of its structural and technological properties.

The design and production of low-sugar foods have become a new concept, as recent data show the effects of high sugar intake on the progression of various diseases, such as diabetes and obesity. As a result, changes in consumer behaviour occur, and demand for healthier products is also increasing [13]. Jams are one of the products with high sugar content, and recent studies have investigated the effects of reducing or removing sucrose from formulations. However, this reduction generally negatively affects viscosity and low-methoxy pectin (LMP), thanks to its gel-forming ability without sucrose, which is suggested as a solution to overcome the issue [14]. After revealing that the current study obtained LMP, the study also investigated its use in the production of low-sugar quince jam. The effects of LMP on jam characteristics and rheology were explored. That makes this study the first to discover the potential of microwave extraction to valorise pickled cabbage waste and to investigate the effects of the obtained LMP on the properties of low-sugar jam.

## 2. Materials and Methods

### 2.1. Materials

A local pickle factory (Bayburt, Türkiye) supplied the fermented cabbage waste. The waste was soaked in distilled water to remove the salt and dried. It was dried at 50 °C using a drying oven till a moisture content of 10% was achieved, and later it was ground. The ground sample was held at −18 °C till analysis. Quince and sugar were purchased from a local store to produce jam.

### 2.2. Chemicals

L-rhamnose, D(+)-galacturonic acid and L(+)-arabinose were purchased from Sigma Aldrich (Merck KGaA, Darmstadt, Germany). Carbazole was supplied by Glentham Life Sciences (Corsham, UK). Folin–Ciocalteu reagent and sodium azide were purchased from Merck (Darmstadt, Germany). Gallic acid was supplied by Apollo Scientific (Manchester, UK). D(+)-xylose, D(+)-glucose and citric acid were purchased from AFG Scientific (Arlington Heights, IL, USA). The other substances were of analytical quality.

### 2.3. Experimental Design

Response Surface Methodology (RSM) was used to examine the optimal extraction conditions. The Design Expert Version 22.0.6 (Stat-Ease) package was used for experimental runs and data processing.

The effects of process variables (microwave power, irradiation time, pH) on pectin yield were examined, and a Box–Behnken experimental design was used to develop mathematical models relating the responses to the variables. The boundary conditions of the process variables and levels are shown in Table 1.

### 2.4. Microwave-Assisted Extraction (MAE) and Measuring Pectin Yield

MAE was conducted in a microwave oven (Arçelik MD 201S, Eskişehir, Türkiye) according to the method of Spinei and Oroian [8], with some modifications. A total of 10 g of the dried pickled cabbage waste was dispersed in distilled water acidified with 0.1 M citric acid solvent at different pHs (1.5, 2.0, 2.5) with a solid–liquid ratio of 1:20 (*w*/*v*). The pH values were determined using a digital pH meter (Mettler Toledo, Greifensee, Switzerland). Three different microwave powers (231, 385 and 539 W) and three different time durations (60, 90 and 120 s) were tested. Following extraction and cooling down, the supernatant was obtained by centrifugation (5000 rpm, 30 min) (Hettich Universal 320R, Kirchlengern, Germany). The supernatant was then treated with ethanol (96%, 1:2 *v*/*v*) overnight at 4 °C. The precipitated pectin was separated by centrifugation (5000 rpm, 25 min) and then 96% ethanol was used for further purification. After dialysing against distilled water for 24 h, the pectin was dried at 50 °C and ground for further analyses. The yields were determined according to the formula (Equation (1)).

(1) $$\mathrm{Pectin}\;\mathrm{yield}\;(\%)=\frac{M}{M_{0}}\times 100$$

*M*: the weight of dried pectin (g), *M*_0_: the initial weight of dry fermented cabbage sample (g).

### 2.5. Determination of Galacturonic Acid Content

GA content was determined with some modifications to the carbazole–sulfuric acid method [15]. The absorbance was read at 520 nm using a UV 1800 spectrophotometer (Shimadzu, Tokyo, Japan). D(+)-galacturonic acid (0–200 μg/mL) was used to prepare the standard calibration curve.

### 2.6. Esterification Degree Analysis

The analysis was performed by applying the titration method according to Panwar et al. [16].

### 2.7. Determination of Molecular Weight

High-Performance Liquid Chromatography (HPLC) (Shimadzu, Tokyo, Japan) coupled with a RID-10A detector was used to measure the molecular weight. A PolySep-GFC-P 6000 (Phenomenex, LC Column 300 × 7.8 mm) (Torrance, CA, USA) column was used for analysis. The sample (0.1 mg/mL) was dissolved in 0.01 M Phosphate Buffered Saline (PBS, pH 7.4) and filtered through a 0.45 µm syringe filter. Injection volume was 100 µL, and 0.01 M PBS (pH 7.4) was used as mobile phase at a flow rate of 0.3 mL/min at 30 °C [17].

### 2.8. Fourier-Transform Infrared Spectroscopy (FTIR) Analysis

The FT-IR spectrum of microwave-extracted pectin (MEP) was obtained using a spectrometer (Nicolet 6700, Thermo Scientific, Waltham, MA, USA). The spectrum was recorded between 400 and 4000 cm^−1^ [18].

### 2.9. Monosaccharide Analysis

Monosaccharide profiling of MEP was performed by HPLC, as reported by Yalmancı et al. [19]. Galacturonic acid, xylose, arabinose, glucose and rhamnose were used as standard sugars.

### 2.10. Scanning Electron Microscopy (SEM) Analysis

The pectin sample was covered with gold and analysed using an FEI Quanta 450 FEG (FEI Technologies Inc., Hillsboro, OR, USA) scanning electron microscope. The scanning was carried out under high vacuum with an acceleration voltage of 5.0 kV [20].

### 2.11. ^1^H NMR Analysis

The ^1^H NMR spectrum of pectin was determined using the method by Rahmani et al. [21] with some modifications. A total of 30 mg of pectin was solubilised in D_2_O, and the analysis was performed at 25 °C. The spectrum was acquired using a 400 MHz Bruker AVANCE AvanCore (Bruker, Reinstetten, Germany).

### 2.12. Determination of Water- and Oil-Holding Capacity

The method described by Xu et al. [22] was used to measure the water- and oil-holding capacities of pectin.

### 2.13. Determination of Emulsion Activity and Stability of Pectin

Emulsion stability (ES) is measured as the percentage of the initial emulsion volume (IEV) that remains as the final emulsion volume (FEV) after a specified period. The emulsifying activity (EA) was determined as the percentage ratio of the emulsion phase volume (VEP) to the total system volume (TVS). Emulsion stability (ES) and emulsion activity (EA) of pectin samples were evaluated using the method of Hosseini et al. [23]. Emulsion stability (ES) was tested at both room temperature and +4 °C (for 1 day and 30 days) and calculated using the formula below (Equation (2)).

(2) $$\mathrm{ES}\;(\%)=\frac{FEV}{EIV}\times 100$$

*FEV*: Final Emulsion Volume; *IEV*: Initial Emulsion Volume.

Meanwhile, EA was measured as follows (Equation (3)).

(3) $$\mathrm{EA}\;(\%)=\frac{VEP}{TVS}\times 100$$

*VEP*: volume of emulsion phase; *TVS*: total volume of the system.

### 2.14. Determination of Total Phenolic Content

The Folin–Ciocalteu calorimetric method was carried out for TPC analysis [16,24]. The results were expressed as mg gallic acid equivalent per gram of pectin (mg GAE/g pectin).

### 2.15. Jam Production

This study investigated the production of low-sugar quince jam using MEP. To test the effect of pectin on jam properties, 2 different control jams (no pectin jam and a jam with commercial apple pectin) were also produced. After being washed, quinces were first precooked for ten to fifteen minutes at 85 °C. pH was adjusted to 3.3  ±  0.1 using a 0.6% proportion of g citric acid/ 100 g dry ingredient. Then, 1% MEP, 0.13% CaCl_2_ and 44% sucrose were added for jams with pectin. Pectin was replaced with 1 g of sucrose in no-pectin jam. Jam production was continued until at least a Brix value of 45% was reached [14] and jam formulations were given as Supplementary Materials.

### 2.16. Jam Chemical Analysis

A refractometer was used to determine total soluble solids (°Brix) at room temperature. The pH value was determined using a digital pH meter (Mettler Toledo, Greifensee, Switzerland). Titratable acidity was determined by the acid–base method: 2 g of the jam sample was homogenised with 50 mL of distilled water, and the mixture was titrated with NaOH in the presence of phenolphthalein until a faint pink colour was observed. Titratable acidity was expressed as % citric acid. TPC was determined following the Folin–Ciocalteu calorimetric method [16,24]. Antioxidant activity was measured using the DPPH assay [25].

### 2.17. Rheological Analysis

The viscoelastic behaviour was determined by analysing storage modulus (G′) and loss modulus (G″) using the HR Discovery rheometer (TA Instruments, New Castle, DE, USA) equipped with a Peltier plate. A parallel-plate geometry system with a 20 mm diameter was used at a measuring gap of 1.0 mm. A strain sweep test was performed over the strain range of 0.01 to 100% to determine the viscoelastic region for all the jam samples. Frequency sweep tests were performed in the angular frequency (ω) range of 0.1–100 rad/s at a constant strain determined by the sweep strain test at 25 °C. The storage modulus (G′), loss modulus (G″), loss tangent (tan *δ*), and complex viscosity were obtained as rheological data [26].

### 2.18. Electrical Consumption and CO_2_ Emission

The environmental effects of the method were measured by calculating electrical consumption (EC) and CO_2_ emission (E_CO2_) (Equations (4) and (5)) [27,28]. The current emission factor of the Turkish electricity grid is approximately 0.436 kg CO_2_/kWh, so the calculation was modified.

EC = *P* × *t* (4)

*P*: Electrical power (kW); *t*: Time (h).

E_CO2_ = *EC* × 0.436 (5)

### 2.19. Statistical Analysis

Data are presented as mean ± SD from triplicate experiments. Pectin samples were compared using ANOVA and Tukey’s test (*p* ≤ 0.05) via IBM SPSS version 22.

## 3. Results and Discussion

### 3.1. Model Fitting

**Figure 1:**
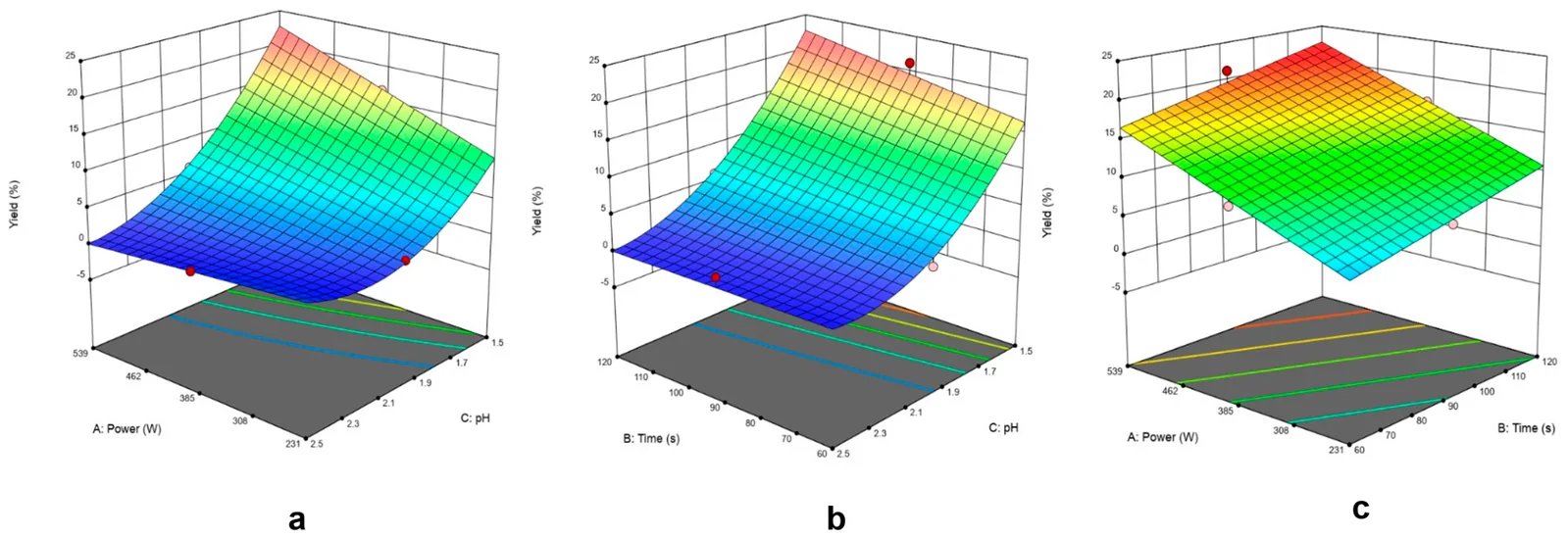
(**a**–**c**) 3D response surface plots showing interactions between process parameters for microwave-assisted extracted of pectin (MEP).

A mathematical model for pectin yield (%) was constructed via multiple linear regression (Table 2) and simplified by removing insignificant terms to produce a second-degree polynomial equation (Equation (6), where A = power, B = time, and C = pH). The model was statistically significant (F = 63.24, *p* < 0.05), with A, B, C, AC, BC, and C^2^ identified as the significant terms. Model reliability was supported by a predicted R^2^ of 0.8833, an adjusted R^2^ of 0.9589 (Table 3), and an adequate precision of 27.5866. The corresponding interactions are illustrated in 3D response surface plots (Figure 1a–c). Optimisation yielded ideal extraction parameters of 539 W microwave power, 116 s, and pH 1.5.

(6) $$\mathrm{Yield}=2.61+2.32A+1.48B-6.08C-3.62AC-1.61BC+4.99C^{2}$$

### 3.2. The Effect of Extraction Variables on Pectin Yield

The ideal conditions for the highest pectin yield were determined to be 539 W, 116 s, and pH 1.5, and the predicted yield was 22.13%. The desirability function was used to determine the optimal conditions, so trials were conducted, and the experimental yield was 22.53%. This yield is comparable to that obtained from other food waste streams as a pectin source. MAE stands as an alternative to conventional acid extraction for pectin utilisation. Gonzalez Serrano et al. [29] studied discarded garlic peels and optimised pectin yield, concluding that MAE provides higher pectin yield (14.50%) compared to conventional heat extraction (12.10%). Sarah et al. [30] investigated coca pod husk as a pectin source using MAE and reported a pectin yield of 21.10% under optimal conditions. In addition, our previous study using conventional extraction also reported 14.14% pectin yield from pickled cabbage waste [11].

The model suggests that all three factors have a significant impact on pectin yield (Figure 1a–c).

pH was identified as the most influential factor, and the highest pectin yield was observed at pH 1.5, as reported in previous studies [21]. This effect can be seen in 3D figures plotting pH versus microwave power or irradiation time (Figure 1a,b). Higher H^+^ concentration increases the hydrolysis of protopectin into pectin, resulting in higher pectin recovery from the sample [27].

Microwave power and processing time were reported in previous studies to be important factors to increase pectin yield. An increase in microwave power and irradiation time loosens the plant cell wall, allowing the solvent to enter the plant matrix [21,22,23,24,25,26,27,28,29,30]. Moreover, increasing microwave power and time when the pH was kept at a low level also elevated the pectin yield, as shown in the 3D plot (Figure 1c).

MAE performance depends largely on how the solvent and biomass interact with microwave energy (their dielectric properties), as well as the nature of the substances being extracted. In the case of pectin, microwave heating is suggested to be particularly beneficial because it speeds up mass transfer and helps dissolve cell wall polysaccharides more effectively [31]. Previous studies report that MAE is an effective, time-saving method for extracting pectin from food waste streams. Although our previous study reported a higher pectin yield (30.32%) using ultrasound-assisted extraction for 45 min [11], the pectin yield of MAE proves the quickness and efficiency of the method.

### 3.3. Physicochemical Characterisation

Galacturonic acid (GalA) is a parameter that determines both the purity and the intended use of pectin. The GalA content of MEP was recorded as 81.28 ± 4.43%, which was higher than the value recommended by the Joint FAO/WHO Experts Committee on Food Additives (JECFA) (at least 65%), and this proves that the MEP could be used as a food additive.

The structure of pectin is composed of linear chains of ᴅ-galacturonic acid, which is interrupted by L-rhamnose with side chains including L-arabinose units. According to Table 4, the monosaccharide composition of MEP was galacturonic acid, glucose, arabinose, xylose, and rhamnose. The GalA was the main monosaccharide with the highest ratio of all monosaccharides, and this also indicated that GalA was the backbone of homogalacturonan and rhamnogalacturonan regions of the MEP. While the main neutral monosaccharides were arabinose and rhamnose, which are associated with the rhamnogalacturonan structure, a low percentage of glucose and xylose might indicate their presence on side chains [32]. Although the monosaccharide compositions of white cabbage pectin were generally consistent with previous studies [11,12,33], differences in the monosaccharide profiles were observed depending on the extraction method. For example, the contents of glucose, xylose, rhamnose, and arabinose were lower (4.70%, 3.65%, 1.65%, and 2.16%, respectively) in pectin obtained by ultrasound-assisted extraction [11]. Moreover, Müller-Maatsch et al. [12] and Westereng et al. [33], who employed acid and ethanol extraction, respectively, reported the presence of galactose and arabinose in addition to the monosaccharides mentioned above. These differences may be attributed to the selective extraction of different pectin fractions by the applied extraction methods rather than to changes in the intrinsic monosaccharide composition of pectin during extraction.

The results indicate that MEP had a DE of 23.89 ± 0.37%, showing a low degree of esterification, which revealed that the pectin samples had low DE and were consistent with a previous study on cabbage. Müller-Maatsch et al. [12] examined various food wastes for their pectin content and compared the pectic material in fresh and sour cabbage. The study concluded that pectin from fresh and sour cabbage had methylation levels of 19% and 12%, respectively. Our previous study using conventional and ultrasound-assisted extraction also confirmed the presence of low-methoxy pectin in pickled cabbage waste [11]. Sandarani [34] stated that solvent choice and extraction method are key factors in pectin properties. For example, studies using citric acid in extraction observed that the pectin obtained had better gelling properties, and in some studies, a higher pectin yield was obtained.

### 3.4. Structural Characterisation

**Figure 2:**
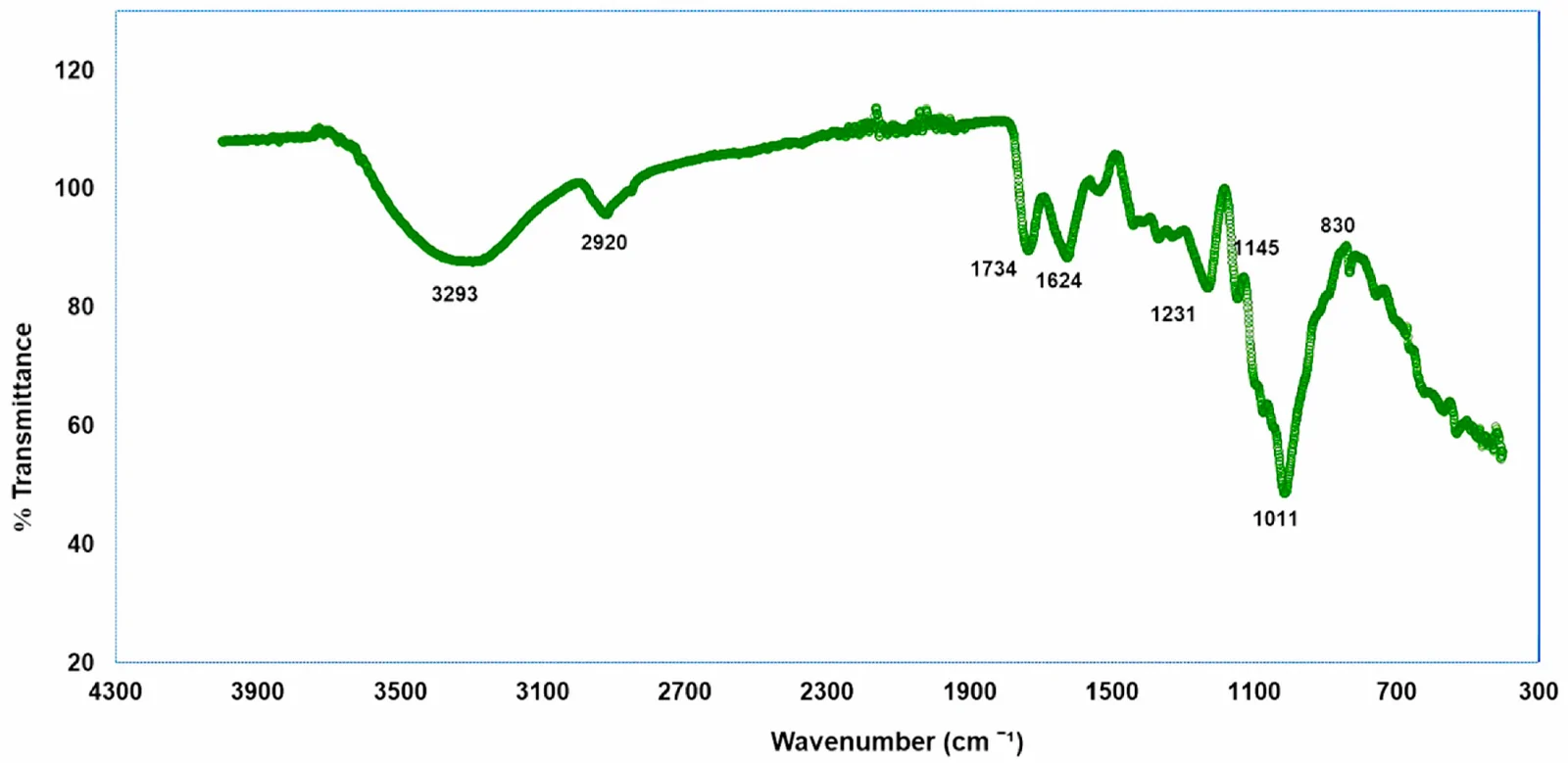
The FTIR spectrum of MEP.

The infrared spectra of MEP are shown in Figure 2. The spectrum pointed out the ‘fingerprint’ area of carbohydrates between 1200 and 950 cm^−1^, which is considered one of the typical bands for the pectin molecule. The absorbance between 950 and 1200 cm^−1^ confirms that MEP is pectin [15,35].

The peaks at 2920 cm^−1^ corresponded to stretching vibrations of C-H bonds in CH, CH2, and CH3 groups. The signals at 1011 cm^−1^ and 1145 cm^−1^ can be assigned to C-O and C-C, and the absorption at 1230 cm^−1^ is ascribed to C-O-C vibrations of glycoside content [36]. The peak at 830 cm^−1^ indicates an α-arabinose pyranose ring [8]. The absorption at 1734 cm^−1^ is attributed to the ester carbonyl groups (C=O), and the intensity at this spectrum is related to the DE. Furthermore, the peak at 1624 was derived from the free carboxyl group (COO^−^), and the decreased intensities of this area indicate the lower degree of esterification [37]. Therefore, this FTIR spectrum also proved the low DE of MEP obtained from chemical analysis. A similar observation was performed by Rahmani et al. [21].

**Figure 3:**
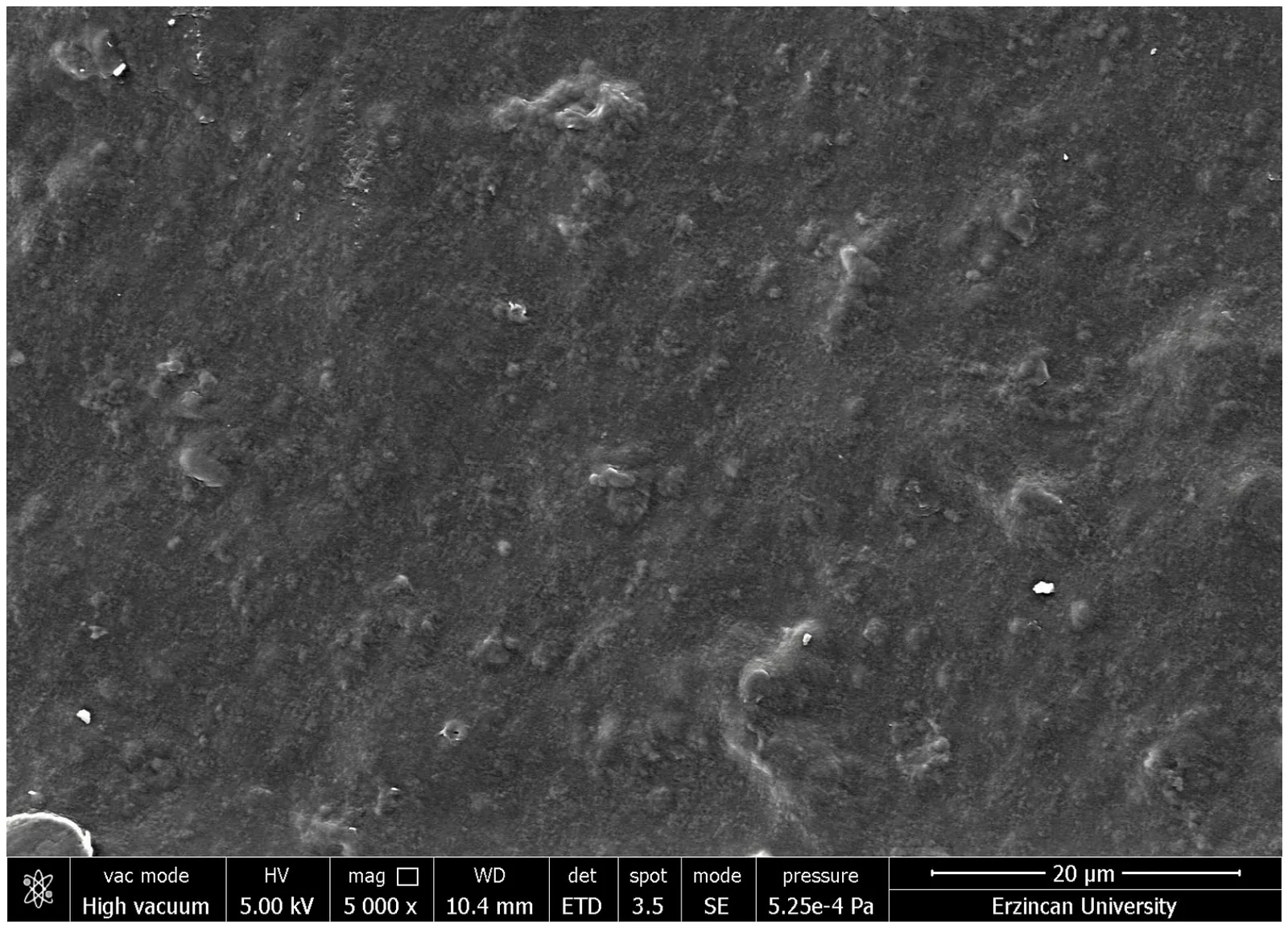
The SEM image of MEP.

Scanning electron microscopy was applied to assess the microstructure characterisation of MEP. According to Figure 3, MEP exhibited relatively clustered and rough surface characteristics, as also observed by Kazemi et al. [36] and Sengar et al. [20]. Furthermore, the drying method affected the morphological characteristics. Since the pectin was dried in a drying oven, thermally induced moisture removal could have resulted in structural shrinkage and partial pore collapse. These changes may have led to a more compact, denser morphology, accompanied by a reduction in the apparent porosity observed in Figure 3. Sudden temperature changes during MAE could also have affected the composition of pectin macromolecules, which in turn led to the observed morphological features. Therefore, this wrinkled surface might have arisen from sudden temperature changes during MAE, and the resulting internal pressure from rapid temperature increases could have caused roughness and aggregated surface characteristics on the pectin [38]. Sun et al. [39] observed similar sheet surface morphology on pectin when citric acid was used for extraction, and they concluded that the acid used for extraction determined the surface morphology characteristics of pectin.

Molecular weight is one of the determinants for industrial utilisation of pectin since it is related to functional properties such as emulsification, encapsulation and viscosity behaviour [40]. In this study, the weight-average molecular weight of pectin was found to be 1.20 ± 0.004 MDa, which was lower than that of fig skin pectin obtained by MAE (6.25 MDa). Gharibzahedi et al. [41] stated that MAE might induce a more complex structure with a high number of GalA units; thus, the molecular weight might be reported as higher than in previous studies [42,43,44]. Kazemi et al. [36] also concluded that the extraction method and conditions are the main determinants of pectin molecular weight, as well as the pectin source.

**Figure 4:**
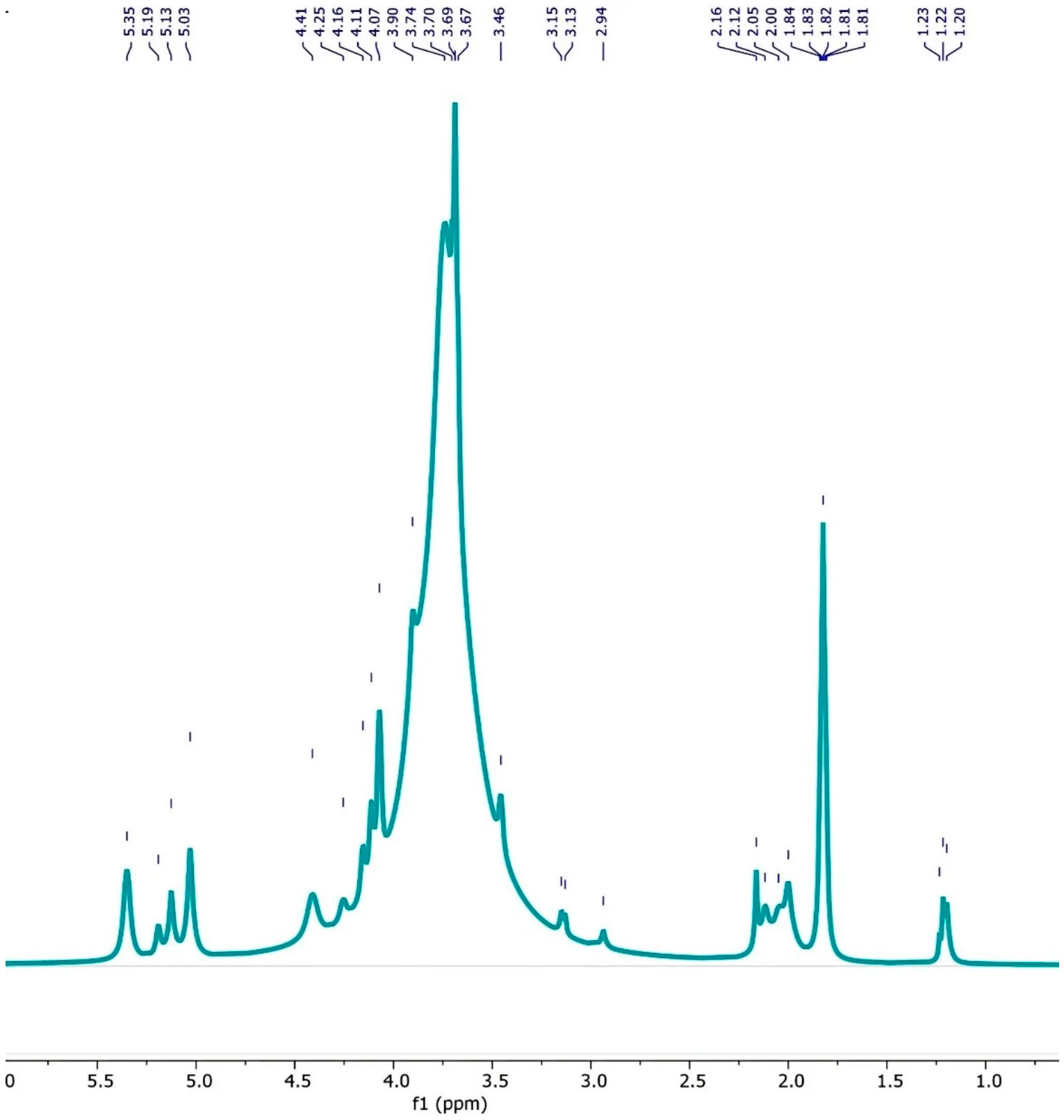
The ^1^H NMR spectrum of MEP.

The ^1^H NMR spectrum of MEP is shown in Figure 4, and the most intense peak was observed at 3.69 ppm, which designates the methoxyl groups associated with the carboxyl groups of GalA. The typical anomeric H-1 proton signals were observed at 5.13 and 5.35 ppm as a result of α-1,4-linked Gal A residues [21]. Furthermore, the signal at 5.03 ppm indicates the H-1 of different types of arabinoses. Similar results were also obtained at 5.15 ppm and 5.40 ppm by Choudhury et al. [45] for *Dillenia indica* fruit pectin. The signals between 4.16 ppm and 4.41 ppm were ascribed to the H-4 proton [46]. The range of signals between 2.0 and 2.16 ppm was attributed to acetyl groups (-COCH_3_) of bonded GalA [42]. The signals at 1.20 ppm, 1.22 ppm, and 1.23 ppm were assigned to the branched and unbranched methyl groups of rhamnose [36]. These observations were also in line with the results from monosaccharide composition.

### 3.5. Water and Oil-Holding Capacity of MEP

The results showed that MEP had a higher water-holding capacity (9.36 ± 3.25 g water/g pectin) than oil-holding capacity (1.18 ± 0.51 g oil/g pectin) (Table 5). The water-holding capacity of MEP was higher than that reported in our previous study using ultrasound-assisted or conventional extraction [11]. This emphasises the positive effect of MAE on pectin’s water-holding capacity. Factors such as the extraction method and pectin porosity can affect water-holding capacity [36], which might explain differences between pectins from the same source. The water- and oil-holding capacities of pectin, in terms of its functions in food systems, improve food structure and impact shelf life. Previous studies have reported that pectin with a low DE has more free carboxyl groups and interacts more with water than pectin with a high DE [47,48].

### 3.6. Emulsion Activity and Stability

**Figure 5:**
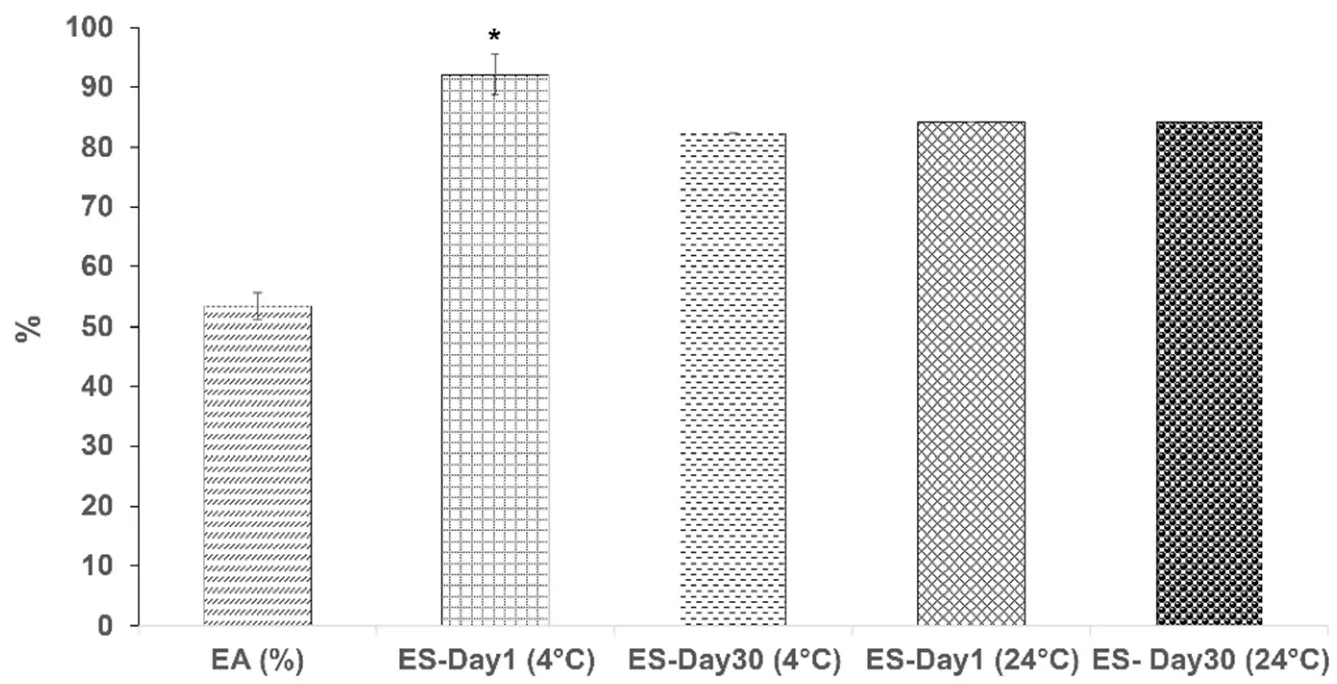
Emulsion activity and stability results of MEP. EA; emulsion activity, ES; emulsion stability. Data are presented as mean ± SD. * Represents the significantly highest value compared to all emulsion stability results. There were no statistically significant differences among the remaining emulsion stability results.

Emulsion activity and stability results are given in Figure 5. MEP had an emulsion activity of 53.43 ± 2.19%. At 4 °C, emulsion stability was recorded as 92.15 ± 3.39 for day 1 and 82.35 ± 0.00 for day 30. At room temperature, pectin showed an emulsion stability of 84.21 ± 0.00 for day 1 and kept it after 30 days of storage. Although the emulsion stored at 4 °C exhibited the highest stability on the first day of storage, this initial advantage progressively diminished, and by the end of 30 days, its stability became comparable to that of the emulsion stored at room temperature. As shown in Figure 5, there is no significant difference between samples stored at 4 °C and 24 °C after 30 days of storage. This might have originated from the structural characteristics of the pectin, since molecular weight, degree of esterification, neutral sugar side chains, and pH can affect the emulsion properties and stability of pectin. It has been stated that high molecular weight contributes to intermolecular interactions and aggregation, which can limit the effective adsorption and organisation of pectin at the oil–water interface [49]. Therefore, the high molecular mass of the pectin may initially have provided an effective protective layer, while prolonged storage may have allowed gradual changes in the organisation of this layer and in droplet–droplet interactions. Consequently, the initial stabilising advantage of 4 °C diminished over time, resulting in an emulsion stability comparable to that observed at room temperature after 30 days. The results also revealed that MEP emulsions were stable at both 4 °C and room temperature, indicating the potential of MEP as an emulsifier in food manufacturing.

As LMP does not require a high sugar content to form a gel in the presence of divalent cations such as calcium, it is a better option for low-sugar or sugar-free products [50]. Considering that, the current study also investigated the effects of MEP on the production of low-sugar jam.

### 3.7. Jam Analysis Results

This study investigated the production of low-sugar quince jam using MEP (MEPJ) and compared it to jam without pectin (NCJ) and jam containing commercial apple pectin (CAPJ). Chemical properties of the produced jams are given in Table 6. The Brix results revealed no significant difference between controls, but MEPJ had a remarkably lower Brix value (*p* < 0.05), which was also lower than the normal quince jam, which had a Brix range of 65.40–66.30 [51], as expected. The pH values were similar in all samples (3.25–3.26) and compatible with the National Turkish Food Regulations (allowed pH:2.8–3.5). Jams produced in the study had low pH values; however, these samples may have had a shorter shelf life due to Brix values below 70 [52]. Regarding titratable acidity, the highest values were observed in the controls as well.

Previous studies have shown that quince is rich in phenolic acids, flavonoids, and organic acids, owing to its antioxidant properties [53,54]. Flavan-3-ols, flavonols, phenolic acids, and anthocyanins were previously determined in quince jam products [55]. The antioxidant activity of the samples was recorded as CAPJ > NCJ > MEPJ (*p* < 0.05). Although the TPC of NCJ was lower than that of a previous study (1.31 mg GAE/g sample) [51], the highest TPC was determined in NCJ. The effects of the quince variety and the jam-making process can account for this discrepancy.

Although NCJ showed the highest total phenolic content, it ranked second in DPPH (%). A Brix value of 45 was targeted and measured during jam production to achieve a low sugar content. It took longer to reach this value in NCJ, and this may have led to release of trapped phenolics in quince, which were recorded as higher total phenolic content.

### 3.8. Rheological Analysis Results

**Figure 6:**
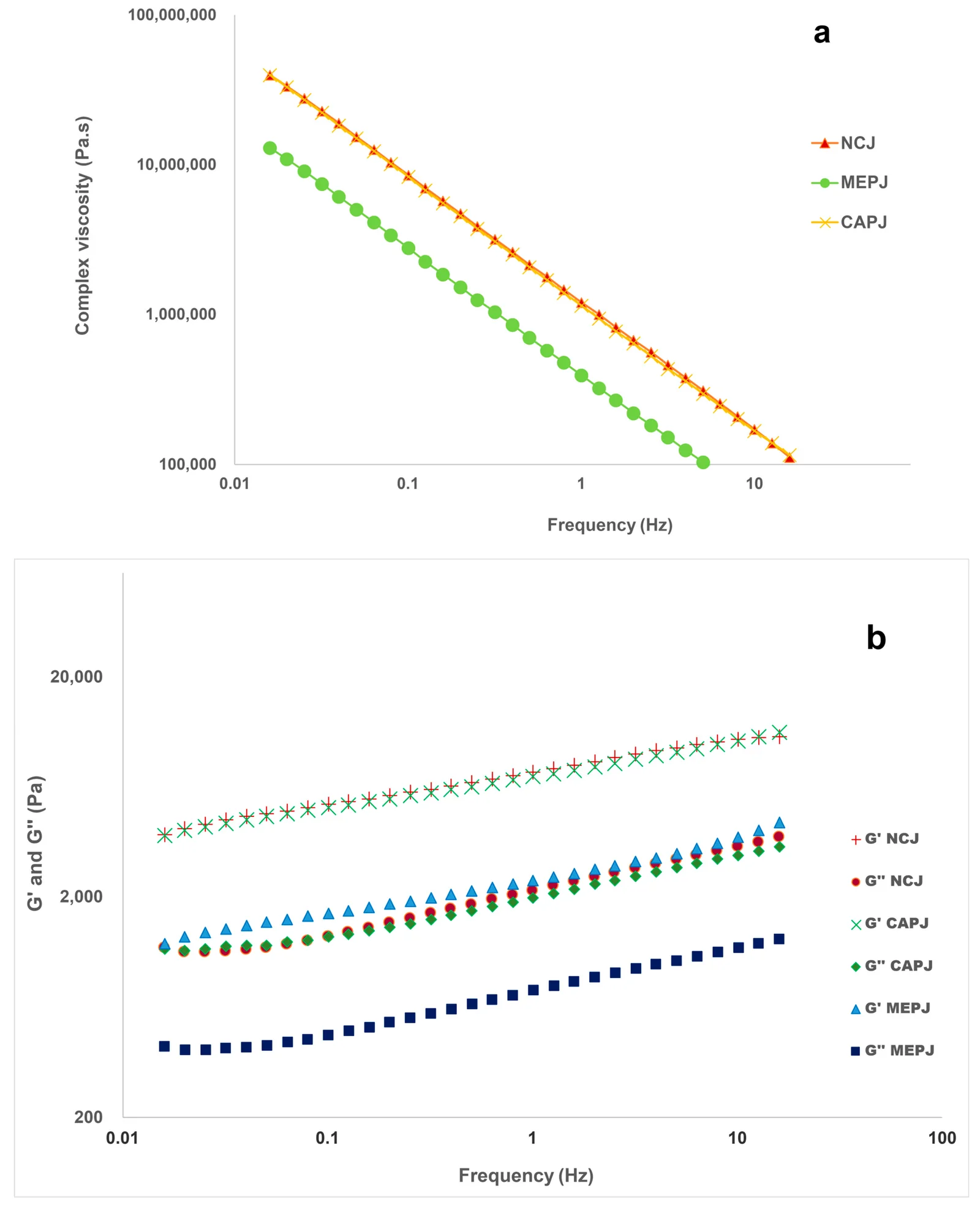

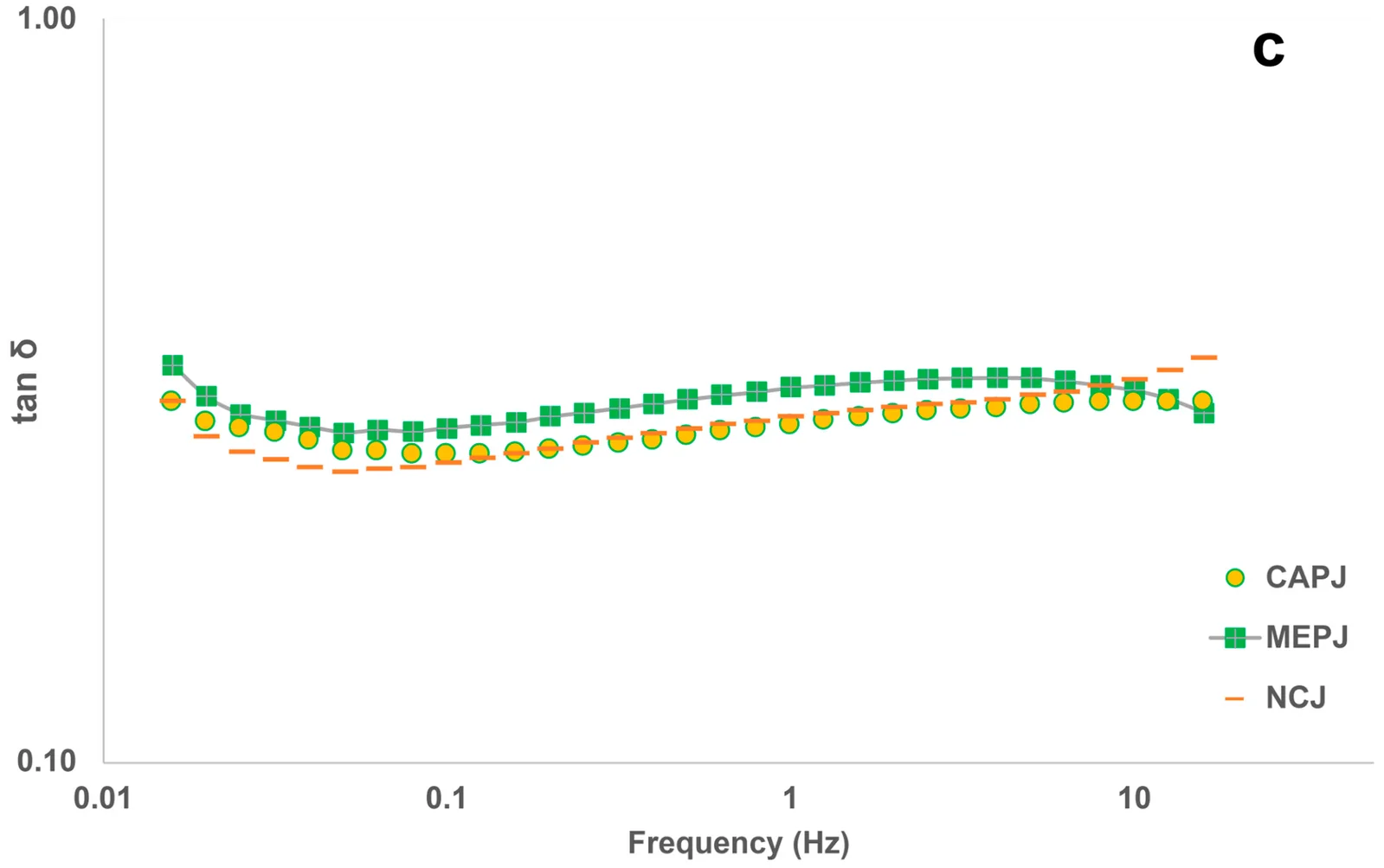
(**a**) Complex viscosity vs. angular frequency of NCJ, CAPJ and MEPJ; (**b**) storage modulus (G′) and loss modulus (G″) vs. angular frequency of NCJ, CAPJ and MEPJ; (**c**) tan *δ* vs. angular frequency of NCJ, CAPJ and MEPJ. NCJ: jam without pectin, CAPJ: jam containing commercial apple pectin, MEPJ: jam containing microwave-assisted pectin.

The viscoelastic properties of jams are significant characteristics to determine the stability and shelf-life of the product; thus, the dynamic elastic properties of NCJ, CAPJ and MEPJ were measured by frequency sweep as a function of complex viscosity, G′, G″ and loss tangent (tan *δ*). The complex viscosity of all jams decreased with increasing frequency, indicating a non-Newtonian flow behaviour (Figure 6a). Furthermore, the complex viscosity of MEPJ was lower than that of CAPJ and NCJ for all angular frequencies, though the intrinsic viscosities of CAPJ and NCJ were almost the same since they overlapped. The slope of the intrinsic viscosity versus angular frequency is also an indicator of gel strength. Nourmohammadi et al. [56] stated that a high level of this slope suggested elastic behaviour of the gel and the ability for weak gel to form, although this behaviour is also influenced by pectin concentration and sugar. In these samples, this situation might have resulted from macromolecular interactions in solution; thus, the jam samples displayed elastic and viscous features at low angular frequency [57]. This type of behaviour is also typical of jams including LMP, and has been reported in previous studies [56,57,58].

The evolution of the storage (G′) and loss (G″) moduli with frequency within the linear viscoelastic regions for quince jam samples (NCJ, CAPJ, MEPJ) are given in Figure 6b. As shown, G′ was found to be higher than G′′ for the whole frequency range for all three samples, which indicates solid-gel-like behaviour, and they were more elastic than viscous. Muñoz-Almagro et al. [14] examined the same behaviour with low-sugar strawberry jam, including LMP. They also noted that the tangled LMP molecules formed clusters via ionic crosslinking between calcium ions and carboxyl groups over time. Then, polymers started to generate aggregates, and the stronger network formed. Thus, as a consequence of the increase in these aggregates and their interactions, the G′ also increased. The MEPJ displayed lower G′ and G″ compared to NCJ and CAPJ. A similar pattern was observed in previous studies, and it is concluded that quince pectin may have characteristics similar to commercial apple pectin, and both pectins are stronger than MEP, as apple pectin exhibits highly viscous shear. Furthermore, it was reported that quince pectin has a higher DE than MEP [59]. Therefore, the addition of MEP to quince jam might result in the dismantling of the original structure and the formation of a new molecular network [60,61].

The loss factor (tan *δ*) demonstrates the comparison of the amount of energy lost during a test cycle to the amount of energy stored during this time [62]. The tan *δ* value of jam samples ranged from 0.24 to 0.34, and the value of MEPJ was slightly higher than that of NCJ and CAPJ, although they were almost in the same range (Figure 6c). Basu et al. [63] stated that decreasing tan δ while increasing total soluble solids indicates an increase in elastic behaviour in jam systems. These values also confirm the gel-like behaviour of jams and their three-dimensional network, and the viscoelastic properties are similar since the chemical bonding for gel-like formation could have occurred [62]. These results align with the study performed by Nourmohammadi et al. [56], who observed similar results (tan δ < 1) with low-sugar sour cherry jam, including 40% sugar.

### 3.9. Energy Consumption and Environmental Impact

Energy consumption and CO_2_ emission for pectin extraction by microwave-assisted method were estimated using Equations (4) and (5). Using the optimised conditions, approximately 2.25 g of pectin was obtained from 10 g of waste material, and the specific energy consumption was calculated as the energy spent per gram of product. As a result, the energy consumption and CO_2_ emission for microwave extraction were found to be 7 Wh/g pectin and 3 g/g pectin, respectively. These values are way lower than those reported in the previous study, which used ultrasound-assisted extraction for pectin derivation [11]. Therefore, it can be concluded that MAE is an energy-efficient and time-saving method to obtain pectin from pickled cabbage waste. Kumari et al. [5] reported similar results for pectin from kinnow peel and noted that MAE is the most effective method among ultrasound, acid, and aqueous extractions regarding pectin yield, energy consumption, and environmental impact.

## 4. Conclusions

The current study uncovered the potential of pickled cabbage waste as a novel pectin source via microwave-assisted extraction. RSM studies revealed that microwave power, time, and pH were significant variables that impacted pectin yield. In addition, MAE enabled us to obtain pectin from pickled cabbage waste in a shorter amount of time compared to ultrasound-assisted and conventional extraction, and the use of a citric acid solution also supported our goal of an environmentally friendly extraction.

As structural characterisation of MEP revealed its low methoxyl content, this study investigated the use of MEP in low-sugar quince jam, in line with current health trends. The results showed that addition of MEP enhanced the viscoelastic properties of jam, as it had previously shown a similar effect with the addition of apple pectin.

This study exemplifies industrial symbiosis by transforming food waste into valuable pectin for low-sugar jam production. The collaboration with a supplier that is also a jam manufacturer creates a distinct pathway for industrial scaling and commercialisation. Moving forward, partnerships with this local business will focus on refining laboratory formulations for factory production and driving market-oriented product development. Collaboration among stakeholders is critical to advancing research and implementing sustainable management practices for food waste valorisation to meet the Sustainable Development Goals.

## Figures and Tables

**Table 1:** Boundary conditions of the process variables of microwave-assisted extraction.

Independent Variables	−1	0	+1
Microwave power (W)	231	385	539
Irradiation time (s)	60	90	120
pH	1.5	2.0	2.5

**Table 2:** ANOVA table for the reduced model.

Source	Sum of Squares	df	Mean Square	F-Value	*p*-Value
Model	524.98	6	87.50	63.24	<0.0001
A—power	43.20	1	43.20	31.22	0.0002
B—time	17.52	1	17.52	12.67	0.0052
C—pH	295.85	1	295.85	213.84	<0.0001
AC	52.42	1	52.42	37.89	0.0001
BC	10.40	1	10.40	7.52	0.0208
C^2^	105.58	1	105.58	76.31	<0.0001
Residual	13.84	10	1.38		
Lack of fit	13.60	6	2.27	38.11	0.0017
Pure error	0.2379	4	0.0595		
Cor total	538.81	16			

**Table 3:** Fit statistics for the reduced quadratic model.

Std. dev.	1.18	R^2^	0.9743
Mean	4.96	Adjusted R^2^	0.9589
C.V. %	23.70	Predicted R^2^	0.8833
		Adeq precision	27.5866

**Table 4:** Characteristics of MEP.

	MEP
Pectin yield (%)	22.53 ± 2.63
Degree of esterification (DE) (%)	23.89 ± 0.37
Total phenolic content (TPC)	2.87 ± 0.28
Monosaccharide composition (%)	
Galacturonic acid (GalA)	81.28 ± 4.43
Arabinose	4.40 ± 0.03
Rhamnose	2.46 ± 0.09
Xylose	5.27 ± 0.03
Glucose	6.60 ± 0.04
Weight-average molecular weight, Mw (MDa)	1.20 ± 0.004

**Table 5:** Water and oil retention capacity results of MEP.

	Water-Holding Capacity (g Water/g Pectin)	Oil-Holding Capacity (g Oil/g Pectin)
MEP	9.36 ± 3.25	1.18 ± 0.51

**Table 6:** Chemical properties of produced jams.

Chemical Properties	NCJ	CAPJ	MEPJ
Brix	47.50 ± 0.50 ^a^	46.83 ± 0.28 ^a^	45.50 ± 0.50 ^b^
pH	3.25 ± 0.01 ^a^	3.25 ± 0.00 ^a^	3.26 ± 0.10 ^a^
Titratable acidity	0.64 ± 0.03 ^ab^	0.68 ± 0.02 ^a^	0.61 ± 0.03 ^b^
DPPH (%)	75.24 ± 0.09 ^b^	78.61 ± 1.11 ^a^	70.66 ± 1.44 ^c^
TPC mg GAE/g wet sample	1.01 ± 0.03 ^a^	0.70 ± 0.03 ^c^	0.81 ± 0.01 ^b^

NCJ: Jam without pectin, CAPJ: jam containing commercial apple pectin, MEPJ: jam containing microwave-assisted pectin. Values are mean  ±  SD. Means with different superscript letters in the same line indicate significant difference (*p*  <  0.05) from each other.

## Data Availability

The raw data supporting the conclusions of this article will be made available by the authors on request.

## Supplementary Materials

The following supporting information can be downloaded at: https://www.mdpi.com/article/10.3390/polym18182200/s1, Table S1: RSM experimental design and obtained responses (pectin yield) for microwave extraction, Table S2: Best of three optimized conditions for microwave extraction, Table S3: Formulations used for the manufacturing of low sucrose quince jams.
