# Supplementary material for: Microwave-Assisted Extraction of Pectin from Pickled Cabbage Waste: Process Optimisation, Characterisation and Application in Low-Sugar Jam
Source: Polymers (Basel). 2026 Sep 9;18(18):2200. doi: 10.3390/polym18182200 (PMC13611718; doi:10.3390/polym18182200)
Supplement: Supplementary file 1 [file polymers-18-02200-s001.zip › polymers-4532921-supplementary.pdf]

Supplementary Table S1 RSM experimental design and obtained responses (pectin yield) for microwave extraction

Run	A:Power (W)	B: Time (s)	C:pH	Pectin yield (%)
1	231	60	2	1.12
2	231	90	1.5	7.13
3	539	90	1.5	21.18
4	385	60	2.5	1.03
5	231	120	2	2.15
6	231	90	2.5	2.05
7	539	60	2	2.31
8	385	90	2	2.22
9	385	90	2	2.75
10	385	60	1.5	9.81
11	539	120	2.0	5.93
12	539	90	2.5	1.62
13	385	90	2	2.50
14	385	90	2	2.13
15	385	120	1.5	16.63
16	385	90	2	2.41
17	385	120	2.5	1.40

Supplementary Table S2 Best of three optimized conditions for microwave extraction

Number	Power	Time	pH	Yield (%)	Desirability	
**1**	**539.000**	**116.812**	**1.506**	**22.132**	**1.000**	**Selected**
2	539.000	114.873	1.524	21.192	1.000	
3	539.000	109.500	1.500	21.641	1.000	

Supplementary Table S3 Formulations used for the manufacturing of low sucrose quince jams

**Ingredients**	**NCJ**	**CAPJ**	**MEPJ**
Quince pulp (g)	54	54	54
Sucrose (g)	45	44	44
Pectin (g)	0	1	1
Citric acid (g)	0.6	0.6	0.6
$CaCl_2$ (g)	0.1	0.1	0.1

LM: low methoxyl; CaCl2: calcium chloride; NCJ: Jam without pectin; CAPJ: Jam containing commercial apple pectin; MEPJ: Jam containing microwave-assisted pectin.
